# Systematic Review and Meta-Analysis of the Prognostic Significance of Neutrophil-Lymphocyte Ratio (NLR) After R0 Gastrectomy for Cancer

**DOI:** 10.1007/s12029-018-0127-y

**Published:** 2018-06-13

**Authors:** Katie L Mellor, Arfon G. M. T. Powell, Wyn G. Lewis

**Affiliations:** 1 Wales Post Graduate Medical and Dental Education Deanery School of Surgery, Cardiff University, Cardiff, CF14 4XW UK; 2 Division of Cancer and Genetics, Cardiff University, University Hospital of Wales, Heath Park, Cardiff, UK

**Keywords:** Gastric cancer, NLR, Neutrophil lymphocyte ratio, Prognosis, Survival

## Abstract

**Purpose:**

A meta-analysis was performed to evaluate the prognostic value of neutrophil-lymphocyte ratio (NLR) in patients undergoing potentially curative gastrectomy for cancer (GC).

**Methods:**

Thomson Reuters Web of Science, Ovid MEDLINE(R) and PUBMED databases were searched for relevant articles using search terms neutrophil-lymphocyte ratio (NLR), GC and survival. Articles reporting overall survival (OS), cancer-specific survival and disease-free survival (DFS), in patients undergoing R0 gastrectomy, were studied.

**Results:**

Articles numbering 365 were identified during the preliminary search, and 10 containing 4164 patients were included in the final review. Most patients were > 60 years of age, male (67%) and 2239 (53.8%) had pT3 disease. The number of NLR dichotomization thresholds reported numbered 7, with 2.00 and 3.00 (*n* = 2) the most common. NLR was associated with poor survival in eight studies with hazard ratios ranging from 1.54 (95% confidence interval (CI) 1.26–1.89) to 2.99 (1.99–4.49). Pooled odds ratio (OR) for OS was 2.31 (1.40–3.83, *p* = 0.001) and for DFS 2.72 (1.14–6.54, *p* = 0.020). Four studies presented T-stage data, OR 1.62 (1.33–1.96, *p* < 0.001).

**Conclusion:**

NLR is an important prognostic indicator associated with both OS and DFS after R0 resection of GC, but the critical level is equivocal.

## Introduction

Going by the numbers, gastric cancer (GC) is the fifth most common worldwide cancer diagnosis, and third commonest cause of cancer-related death, accounting for some one million (951,000) new annual diagnoses and 723,000 deaths. [1] Despite earlier diagnosis and improved treatment, nearly one-third of patients undergoing potentially curative surgery experience disease recurrence [2, 3] and new prognostic biomarkers would therefore be very welcome.

The systemic inflammatory response (SIR) is a complex bio-system comprising humoral and cellular components that protect the host from harmful pathogens, and a range of SIR biomarkers has been reported to be associated with poor outcomes in gastric cancer. [2, 4] One such biomarker is the neutrophil to lymphocyte ratio (NLR), based on measurement of the hosts’ circulating immune cells. Reports have associated the NLR with poor outcome; however, studies were not exclusive to patients undergoing potentially curative resection and the value of NLR in this patient cohort is unclear. Moreover, NLR’s value in guiding the use of adjuvant treatment following potentially curative surgery is uncertain. This systematic review and meta-analysis were therefore performed to estimate the prognostic value of NLR in patients undergoing potentially curative gastrectomy and to identify any correlation with histo-pathological T-stage.

## Methods

### Search Protocol

The outcome measures chosen were overall (OS) and disease-free survival (DFS). Thomson Reuters Web of Science, Ovid MEDLINE(R) and PUBMED databases were searched for relevant articles published between 1990 and October 2017. The following search terms were used: (neutrophil lymphocyte ratio OR neutrophil-lymphocyte ratio OR neutrophil-lymphocyte OR neutrophil-lymphocyte-ratio OR NLR) AND (gastr* OR gastric OR stomach) AND (carcinoma OR adenocarcinoma OR malig* OR malignancy OR tumour OR tumor OR neoplasms).

### Study selection (inclusion and exclusion criteria)

All original scientific articles were considered for inclusion. Reviews and book chapters were excluded, as were texts written in languages other than English. Reports including survival analysis of patients who did not undergo surgery with curative intent were excluded. Only studies related to the association between NLR and survival in patients undergoing potentially curative resection for gastric cancer were included in the systematic review. Only studies reporting extractable data related to OS or DFS were included in the meta-analysis.

### Data Extraction

Two independent reviewers applied the inclusion criteria to study abstracts and selected full papers for data analysis. Full-text manuscript data was obtained by author (KM), and 50% of articles underwent independent review (AP), with discrepancies verified by consensus. For each study, baseline data (author, year of publication, country, study period, total number of patients, gender, pTNM stage, neoadjuvant chemotherapy, adjuvant chemotherapy and NLR categorisation thresholds) were recorded. Outcomes were described as odds ratios (OR) with 95% confidence intervals. Where these were not reported, the methods described by Parmar and Rogers were used to extract data from Kaplan–Meier curves, or percentage survival. [5, 6]

### Quality Analysis

This meta-analysis was conducted in accordance with the preferred reporting items for systematic reviews and meta-analyses (PRISMA) guidelines. [7] The quality of the studies was measured using the Newcastle-Ottawa scale, assessing the methodological quality of non-randomised cohort studies for meta-analyses. Studies were judged by two independent assessors, using a nine-point scale comprising analysis on the selection of the study group, the comparability of cohorts and the ascertainment of outcome. Scores above 6 points were taken to denote studies of high methodological quality and were included in the meta-analysis. Studies were excluded if methodological quality was poor (Newcastle-Ottawa scores < 7). [8]

### Statistical Analysis

Statistical analyses were performed using RevMan statistical package (Review Manager (RevMan) Version 5.3. Copenhagen: The Nordic Cochrane Centre, The Cochrane Collaboration, 2014). Heterogeneity between studies was measured by calculating the *I*^2^ statistic that was calculated for an objective measure of heterogeneity. A fixed-effect meta-analysis was performed in all cases, and where there was appreciable heterogeneity (*I*^2^ > 50% or Chi-squared *p* value < 0.10), a random-effect model was used. Corresponding funnel plots of Ln standard error as a function of effect size were used to examine the effect of publication bias visually and were statistically tested using Eggers test; *p* values > 0.05 were taken as indicative of no publication bias. For meta-analysis, absolute numbers of deaths, recurrences and pT-stages in the high and low NLR cohorts were extracted.

## Results

**Fig. 1:**
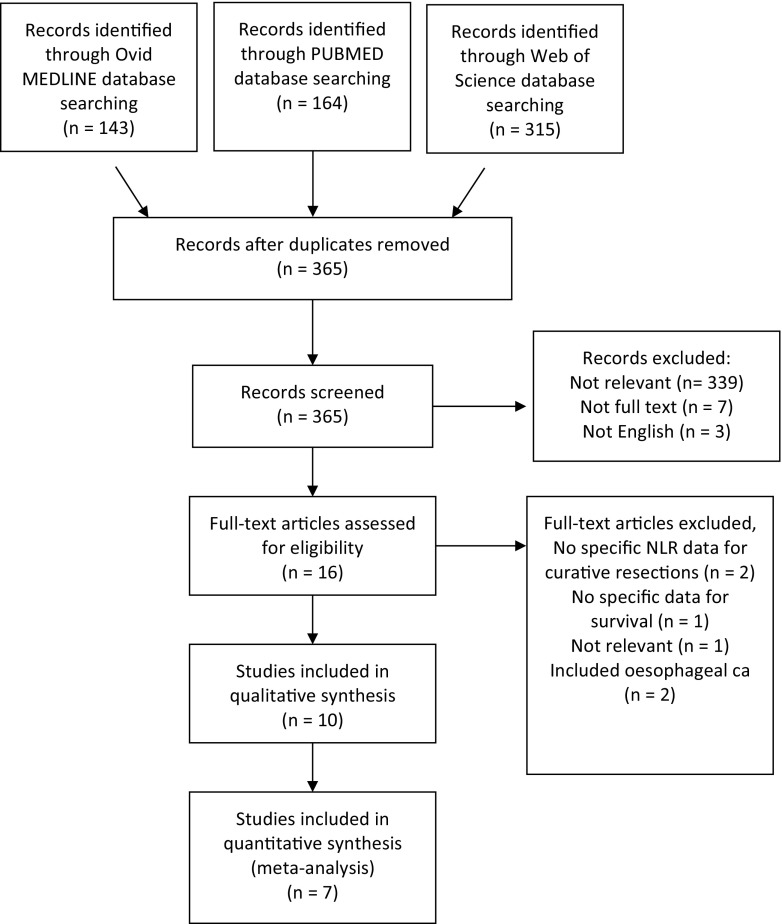
Flowchart of literature selection

The initial electronic search yielded 365 studies, of which 349 were excluded based on abstract content (Fig. 1). Three hundred and thirty-nine were not relevant, seven were not available as full texts, and three were not English language manuscripts. Of the 16 full text manuscripts evaluated, two did not include specific data for curative resections, one did not report survival data, one was not relevant to the study topic and two included data related to oesophageal cancer. Consequently, ten studies were included for qualitative analysis (Table 1) [2, 9–17]. The median Newcastle-Ottawa quality score for these studies was seven (range 7–8). All studies were retrospective cohort studies of one or more regional institutions representing level IV evidence.

**Table 1:** Baseline data on included studies

Author	Year	Country	Study period	No. of patients	Age (years)	M/F ratio	AJCC TNM stage	Chemotherapy	Study design	Evidence level	N-O score
Dutta	2012	UK	1996–2009	120	Median > 65	1.9:1	I = 56 (47%) II = 27 (23%) III = 37 (31%)	NA—38.3% A—not stated	Retrospective	IV	7
Fang	2017	China	2006–2010	190	Median > 60	2.6:1	II = 65 (34%) III = 63 (33%) IV = 62 (33%)	NA—excluded A—not stated	Retrospective	IV	7
Jiang	2014	China	2005–2007	377	Median 64	2.0:1	I = 37 (10%) II = 99 (26%) III = 241 (64%)	NA—excluded A—58.1%	Retrospective	IV	8
Jung	2011	Korea	2004–2007	232^a^	Median 63	Not stated	III = 138 (59%) IV = 94 (32%)	NA—excluded A—not stated	Retrospective	IV	7
Lieto	2017	Italy	2000–2015	297^a^	Not stated	1.4:1	I = 107 (36%) II = 122 (41%) III =66 (22%) IV = 2 (0%)	NA—9.1% A—63.6%	Retrospective	IV	8
Liu	2017	China	2000–2012	1056	Mean 58	2.1:1	I = 194 (18%) II = 266 (25%) III = 596 (56%)	NA—excluded A—suitable stage II–III	Retrospective	IV	7
Mohri	2016	Japan	2000–2011	404	Median 67	2.3:1	I = 260 (64%) II = 70 (17%) III = 74 (18%)	NA—not stated A—not stated	Retrospective	IV	7
Powell	2017	U.K.	2004–2016	291	Median 69	2.0:1	I = 79 (27%) II = 92 (32%) III = 120 (41%)	NA—15.5% A—21.0%	Retrospective	IV	7
Sun	2016	China	2000–2012	873	Median 59	2.1:1	I = 108 (12%) II = 185 (21%) III = 580 (66%)	NA—excluded A—66.7%	Retrospective	IV	7
Wang	2011	China	2006–2009	324	Not stated	2.3:1	III = 324 (100%)	NA—excluded A—64.8%	Retrospective	IV	7

^a^Only data for curative resections extracted

*NA* neoadjuvant, *A* adjuvant, *N-O* Newcastle-Ottawa

The ten studies included a total of 4164 patients with a mean sample size of 418 (range 120–1056). The majority of patients were aged 60 years or older, with an age range of 19 to 89 years and were predominantly male (mean M/F ratio 2.1:1) (Table 1).

Seven studies included patients with pTNM stage I–III disease, and three also including patients with stage IV cancer, all undergoing potentially curative resection (Table 1). The majority of study patients had pT3 disease (*n* = 2239, 53.8%), with fewer patients reported to have locally confined disease (pT1 = 841, 20.2% and pT2 = 926, 22.2%) and 3.8% reported to have pT4 tumours (*n* = 158). Neoadjuvant chemotherapy was given in three of the studies [2, 9, 13] and post-operative chemotherapy was administered to patients in six of the studies. [2, 11, 13, 14, 16, 17] The proportions of patients receiving post-operative chemotherapy ranged from 21.0 to 66.7%.

The NLR critical values reported varied between studies with the commonest value 2.00 and 3.00 (*n* = 2). Six studies reported an association between high NLR and poor overall survival, [10, 11, 14–17] two studies reported an association between NLR and disease-free survival [12, 13] and one study reported no association with cancer-specific survival [10] (Table 2). Four studies reported an association between NLR and advanced pT-stage. [10, 11, 14, 15]

**Table 2:** Baseline data on included studies

Author	Year	Outcome measure	NLR cut-off	5-year survival	Justification of cut-off value	Hazard ratio (confidence interval)	Association with survival
Dutta	2012	CSS	< 2.5—low 2.5–5—intermediate > 5—high	N/A	Not stated	1.19 (0.76–1.87)	No association (*p* = 0.454)
Fang^9^	2017	OS	< 2—low ≥ 2—high	89.5% 76.9%	Median values	2.32 (1.08–5.02)	High NLR, poorer survival (*p* = 0.032)
Jiang	2014	OS	< 1.44—low ≥ 1.44—high	63.2% 36.6%	ROC curve analysis	1.60 (1.05–2.44)	High NLR, poorer survival (*p* = 0.030)
Jung	2011	DFS	< 3—low ≥ 3—high	44.1% 13.0%	Quartiles	1.65 (1.09–2.52)	High NLR, poorer survival (*p* = 0.019)
Lieto	2017	DFS	≤ 3.22—low > 3.22—high	72.6% 30.5%	Maximum log rank statistics	2.99 (1.99–4.49)	High NLR, poorer survival (*p* < 0.001)
Liu	2017	OS	< 2—low ≥ 2—high	62.0% 47.0%	Previous study	1.54 (1.26–1.89)	High NLR, poorer survival (*p* < 0.001)
Mohri	2016	OS	≤ 3—low > 3—high	N/A	ROC curve analysis	2.91 (1.71–4.94)	High NLR, poorer survival (*p* < 0.001)
Powell	2017	DFS and OS	≤ 5.5—low > 5.5—high	70.7%^a^ 59.1%	Previous study	^a^1.43 (0.78–2.65) ^b^1.01 (0.58–1.76)	NLR not associated with survival (*p* = 0.249^a^ and *p* = 0.975^b^)
Sun	2016	OS	< 2.3—low ≥ 2.3—high	85.1% 57.0%	ROC curve analysis	1.66 (1.39–1.99)	High NLR, poorer survival (*p* = 0.001)
Wang	2011	OS	≤ 5—low > 5—high	N/A	Previous study	2.47 (1.21–5.05)	High NLR, poorer survival (*p* = 0.013)

*CSS* cancer-specific survival, *DFS* disease-free survival, *OS* overall survival, *N/A* not available

^a^Data for DFS

^b^Data for OS

### Relationship Between NLR and Overall Survival

**Fig. 2:**
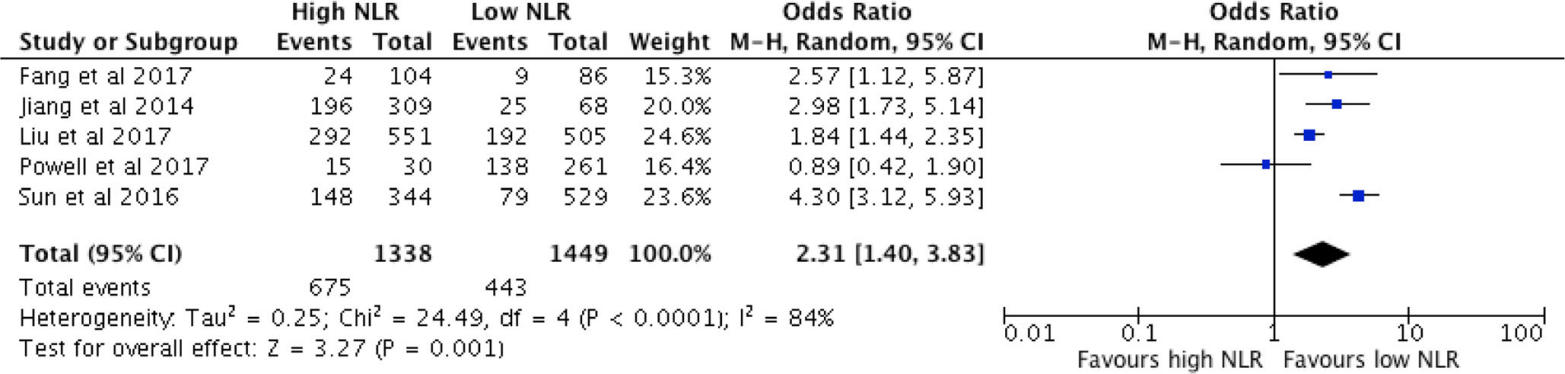
Association between high NLR and overall survival (pooled analysis)

Five studies reported data related to overall survival in 2787 patients undergoing potentially curative R0 gastrectomy. The pooled odds ratio was 2.31 (95% CI 1.40–3.83, *p* = 0.001). Significant study heterogeneity was observed (χ^2^ = 24.49, *df* 4, *p* < 0.0001, *I*^2^ = 84%). The Forest plot for these results is shown in Fig. 2.

#### Relationship Between NLR and Disease-Free Survival

**Fig. 3:**
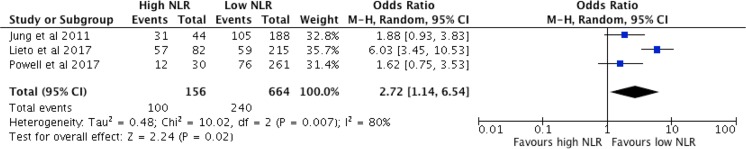
Association between high NLR and disease-free survival (pooled analysis)

Three studies reported data related to NLR and disease-free survival, comprising a total of 820 patients undergoing potentially curative gastrectomy. The pooled odds ratio was 2.72 (95% CI 1.14–6.54, *p* = 0.020, Fig. 3). Once again, significant study heterogeneity was observed (χ^2^ = 10.02, *df* 2, *p* = 0.007, *I*^2^ = 80%).

#### Relationship Between NLR and pT Stage

**Fig. 4:**
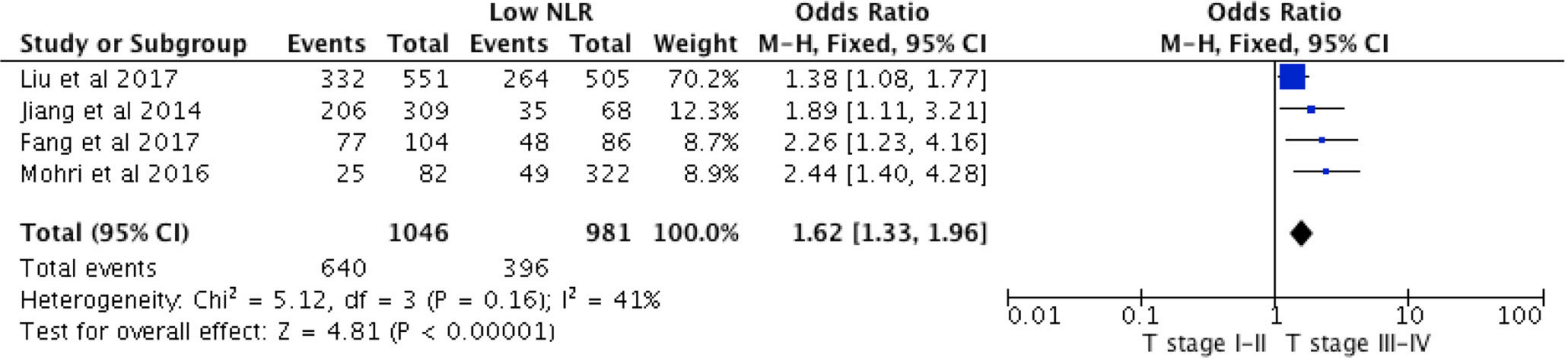
Association between high NLR and T-stage (pooled analysis)

Four studies reported data related to pT stage comprising a total of 2027 patients. The pooled odds ratio was 1.62 (95% CI 1.33–1.96, *p* < 0.001, Fig. 4). No heterogeneity was observed in this cohort (χ^2^ = 5.12, *df* 3 *p* = 0.160, *I*^2^ = 41%).

## Discussion

This meta-analysis is the first to examine exclusively the prognostic value of NLR in patients undergoing potentially curative R0 gastrectomy for cancer. The principal findings were that no fewer than 40% of patients had an elevated pre-operative NLR, in keeping with other gastric cancer meta-analyses (range 36.5–59.8%). [3, 18–20] This patient cohort was over 50% more likely to have higher pT stage, which was associated with over two-fold risk of disease recurrence, and commensurately poorer long-term cumulative survival. Yet, the threshold for NLR dichotomization varied across eight of the studies analysed, threatening any argument for incorporating NLR into a core prognostic algorithm. But such strong predictive associations suggest that the systemic inflammatory response has the potential to be an important therapeutic biomarker, meriting targeted research.

The NLR has been studied in a variety of solid cancers [21] including breast [22], colorectal [23], esophageal [24], lung [25] and pancreas [26]. Reported pooled hazard ratios for NLR and OS have ranged from 1.40 in oesophageal cancer [24] to 2.61 in pancreatic cancer [26], compared with 2.31 in this study. The reason for this is unclear, but may reflect the different dichotomization thresholds reported related to the spectrum of cancers studied. Here, 8 of 10 studies used different dichotomization thresholds; similarly, 9 of 11 reported in breast cancer, 4 of 16 in colorectal cancer, 6 of 7 in oesophageal cancer, 10 of 15 in lung cancer and 4 of 11 in pancreatic cancer. This heterogeneity in dichotomization thresholds is a significant limiting factor in comparing findings between cancer types as well as individual articles grouped by anatomical site, and efforts to standardise reporting of NLR methodology are desirable to facilitate comparative research.

NLR is one among a spectrum of seven inflammation-based prognostic biomarkers associated with poor survival in gastric cancer [2], including CRP, albumin, Glasgow prognostic score, platelet-lymphocyte ratio, neutrophil-platelet score and lymphocyte-monocyte ratio. Inflammation has been proven to play an important role in the development and/or progression of several cancers due to carcinogenesis promotion. [27, 28] Moreover, a meta-analysis of 2.3 million individuals reported a 25% reduction in the risk of gastric cancer associated with NSAID use. [29] Any reasonable observer would surely agree that attenuating the systemic inflammatory response by means of non-steroidal anti-inflammatory drug (NSAID) therapy holds promise. Unfortunately, given the potential adverse side effects associated with routine NSAID use, their indiscriminate peri-operative use would carry a palpable and significant risk. Identifying responders who would benefit the most is therefore an important dilemma to which NLR may hold the key.

This meta-analysis has a number of inherent limitations. Heterogeneity existed between studies, which may be explained by a number of factors, not least the variation in NLR critical values and patient characteristics including disease stage, age and treatment. All studies analysed were retrospective in methodology, and cohort in nature, providing pooled rather than individual patient data. Not all reported extractable data, limiting the number available for analysis. NLR has been described as a possible prognostic marker in other diseases such as the acute coronary syndrome, [30] and as such, the influence of NLR may not necessarily be cancer specific, and attenuating the systemic inflammatory response may promote survival irrespective of the effect of malignancy, which is an arena that requires clarification. In contrast, the study has several strengths. Similar TNM stages and chemotherapy rates to other meta-cohorts strengthen the reliability of the findings. Previous meta-analyses included a heterogeneous mixture of treatment intents, including both potentially curative as well as palliative treatments, and this meta-analysis is the first to exclusively examine NLR’s prognostic significance after potentially curative R0 gastrectomy. The clinical challenge is to identify which patients who have undergone potentially curative resection might benefit from adjuvant therapies because of their higher risk of disease recurrence, regardless of pTNM stage.

In conclusion, despite improvements in staging and surgical technique, approximately one third of patients who undergo potentially curative gastrectomy for cancer will suffer disease recurrence. [31]_._ NLR, derived and calculated from absolute counts of serum lymphocytes and neutrophils, is routinely performed during pre-operative full blood count work-up, and is therefore not only readily available but also inexpensive. Incorporating NLR into MDT algorithms to refine and plan treatment strategies and predict prognosis is currently limited by the variety and inconsistencies in reported dichotomization thresholds. Before this can be achieved, an adequately powered study comparing critical dichotomisation or categorisation threshold is needed to identify a pragmatic optimal critical ratio. Finally, further work should focus on establishing the prognostic value of NLR in patients suitable for potentially curative gastric cancer surgery followed by planned adjuvant anti-inflammatory treatment.
